# Cognitive performance among diverse Asian American subgroups: exploring the role of nativity, language, and education

**DOI:** 10.1038/s44400-026-00108-5

**Published:** 2026-07-03

**Authors:** Alexander Ivan B. Posis, L. Paloma Rojas-Saunero, Simone Subedi, Kristen M. George, Oanh L. Meyer, Alyssa M. Weakley, Maria Rosario Araneta, Paola Gilsanz, María M. Corrada, Rachel A. Whitmer

**Affiliations:** 1 Department of Public Health Sciences, University of California, Davis, Davis, CA USA; 2 Department of Epidemiology, Fielding School of Public Health, University of California, Los Angeles, Los Angeles, CA USA; 3 Department of Neurology, University of California, Davis, Sacramento, CA USA; 4 Department of Family Medicine, School of Medicine, University of California, San Diego, La Jolla, CA USA; 5 Herbert Wertheim School of Public Health and Human Longevity Science, University of California, San Diego, La Jolla, CA USA; 6 Kaiser Permanente Northern California Division of Research, Pleasanton, CA USA; 7 Department of Neurology, University of California, Irvine, Irvine, CA USA; 8 Department of Epidemiology and Biostatistics, University of California, Irvine, Irvine, CA USA

**Keywords:** Neuroscience, Psychology, Psychology

## Abstract

Research often aggregates Asian American, Native Hawaiian, and Pacific Islander (AANHPI) individuals into one group, obscuring possible differences in factors related to cognition. This study included 875 individuals self-identified as AANHPI from the Kaiser Healthy Aging and Diverse Life Experiences (KHANDLE) and *LifeAfter90* studies. Executive function (EF) and verbal episodic memory (VM) were repeatedly measured. Analysis of covariance compared baseline differences while linear mixed-effects models examined cognitive trajectories. We tested moderation by nativity, first language, and education. The average±SD age of participants was 82 ± 9.6 years (47% Chinese, 17% Filipino, 21% Japanese, 15% NHPI/Other). Filipino participants had the lowest baseline EF compared to other subgroups. Relative to Chinese participants, Filipino and Japanese participants had faster EF declines. Nativity and education moderated associations; US-born participants had higher baseline EF but faster decline, and participants with ≥college education had faster EF decline. First language did not moderate associations. Results highlight potential heterogeneity in cognition across AANHPI ethnic subgroups. Differences may be moderated by nativity and education.

## Introduction

The Asian American, Native Hawaiian, and Pacific Islander (AANHPI) population is among the fastest growing groups in the United States (US)^1^, projected to increase from 7.7% of the total US population in 2025 to 11.8% in 2060^2^. Despite their growing representation in the US population and increasing prevalence of dementia with older age, little is known regarding differences in the rates of cognitive aging over time, particularly within AANHPI ethnic subgroups^3–5^. Limited disaggregated AANHPI data are available for dementia risk, which suggest that Filipino Americans may be at greater risk compared with other AANHPI subgroups^3,6^, but none are available to examine aspects of cognitive function. Furthermore, current research typically aggregates AANHPI into a single group, which may obscure potential differences in exposures associated with cognitive impairment as well as differences in cognitive function^7–11^.

Differences in cognitive performance may arise for several reasons such as acculturation. Greater acculturation can lend to positive dementia outcomes such as greater healthcare utilization^12^. Several related factors include nativity (the location of an individual’s birth) and language spoken^13–22^. For example, a 2023 survey found that 54% of the AANHPI population had immigrated to the US^1^, albeit with large differences in immigration timing and patterns across subgroups. Cognitive function and dementia risk may differ by nativity^17,22^. Educational attainment also differs across AANHPI groups^1^ and is a risk factor for dementia^6^. Most studies on the impact of acculturation on cognitive function are among Hispanic/Latino populations^13–18^. While there are few inclusive of AANHPI populations, prior research aggregates AANHPI into one group and not assessed as separate subgroups^19–22^.

Limited studies on cognitive health-related differences between AANHPI subgroups can be partly attributed to the Healthy Immigrant Effect or Model Minority Myth, which posits that AANHPI are well-off and not in need of health-related intervention^23–26^. These misconceptions may portray AANHPI as a monolithic group, reinforcing structural factors to allow aggregated data reporting to persist^7–11^. Aggregated data limits our ability to make evidence-based decisions on policy and interventions focused on minimizing the negative impact of cognitive decline in AANHPI communities, and, thus, all racial and ethnic groups^7–11^. Disaggregated data are important because cognitive testing may be sensitive to early subtle changes and/or provides a window into cognitive aging transitions, potentially allowing for timely and appropriately targeted intervention^27,28^.

Our objective was to compare baseline cognitive function and longitudinal cognitive change across AANHPI subgroups living in Northern California. Given potential differences by level of acculturation, we tested for effect modification by nativity, language, and educational attainment. We hypothesized differences in cognitive function and decline across AANHPI subgroups. We also hypothesized that these differences would vary by nativity, English as a first language, and educational attainment.

## Results

### Participant characteristics

Descriptive characteristics are presented in Table 1. Of 875 AANHPI participants, 47% self-identified as Chinese, 17% Filipino, 21% Japanese, 15% NHPI/Other (see Supplementary Table S1 for disaggregated information on this group category). The average age at baseline was 82 ± 9.6 years. Most participants were women (55.9%) and had at least a college level of education (59.1%). Of the 38.9% of participants who reported immigrating to the US, the average age at immigration was 26.8 ± 12.5 years.

**Table 1:** Participant characteristics by Asian American subgroup

Characteristic		Chinese (*N* = 414)	Filipino (*N* = 145)	Japanese (*N* = 185)	NHPI/Other (*N* = 131)	Overall (*N* = 875)
Age	Mean (SD)	81.1 (9.67)	81.0 (9.32)	84.3 (9.20)	82.4 (9.40)	82.0 (9.55)
Gender	Women	211 (51.0%)	92 (63.4%)	117 (63.2%)	69 (52.7%)	489 (55.9%)
	Men	203 (49.0%)	53 (36.6%)	68 (36.8%)	62 (47.3%)	386 (44.1%)
Education	≤High School/GED	54 (13.0%)	11 (7.6%)	18 (9.7%)	25 (19.1%)	108 (12.3%)
	Some College or Tech/Trade School	108 (26.1%)	43 (29.7%)	57 (30.8%)	40 (30.5%)	248 (28.3%)
	College	136 (32.9%)	73 (50.3%)	61 (33.0%)	29 (22.1%)	299 (34.2%)
	Graduate School	116 (28.0%)	18 (12.4%)	49 (26.5%)	35 (26.7%)	218 (24.9%)
	Missing	*0 (0%)*	*0 (0%)*	*0 (0%)*	*2 (1.5%)*	*2 (0.2%)*
Nativity	Not US born	141 (34.1%)	105 (72.4%)	25 (13.5%)	69 (52.7%)	340 (38.9%)
	US born	273 (65.9%)	40 (27.6%)	160 (86.5%)	62 (47.3%)	535 (61.1%)
First language spoken	English or English and another language	97 (23.4%)	30 (20.7%)	75 (40.5%)	50 (38.2%)	252 (28.8%)
	Not English	224 (54.1%)	80 (55.2%)	79 (42.7%)	54 (41.2%)	437 (49.9%)
	*Missing*	*93 (22.5%)*	*35 (24.1%)*	*31 (16.8%)*	*27 (20.6%)*	*186 (21.3%)*
% life years in the US	Mean (SD)	72.6 (14.4)	61.3 (12.1)	71.3 (8.90)	65.8 (12.6)	67.6 (13.9)
	*Missing*	*273 (65.9%)*	*40 (27.6%)*	*160 (86.5%)*	*62 (47.3%)*	*535 (61.1%)*
Unstandardized SENAS EF	Mean (SD)	−0.10 (0.77)	−0.35 (0.71)	0.02 (0.81)	−0.16 (0.76)	−0.13 (0.77)
Unstandardized SENAS VEM	Mean (SD)	−0.02 (1.01)	0.003 (0.88)	−0.15 (0.97)	−0.18 (0.96)	−0.07 (0.97)
Cohort	KHANDLE	259 (62.6%)	93 (64.1%)	88 (47.6%)	71 (54.2%)	511 (58.4%)
	LifeAfter90	155 (37.4%)	52 (35.9%)	97 (52.4%)	60 (45.8%)	364 (41.6%)

When comparing across AANHPI subgroups, Filipino participants were the youngest (81.0 ± 9.3 years), and had the oldest average age at immigration (32.1 ± 11.8 years). They also had the highest proportion of women (63.4%), college education (50.3%), participants not born in the US (72.4%), and participants who reported not speaking English as their first language (55.2%) compared with other subgroups. Japanese participants had the highest proportion of participants born in the US (86.5%) and having English as their first language (40.5%). Chinese participants had the highest proportion with a graduate school education (28.0%) and the youngest average age at immigration (22.3 ± 12.4 years). NHPI/Other participants had the greatest proportion of participants with some college/tech or trade school level of education or less (49.6%). Of the 340 who reported immigrating to the US, Filipino participants had the lowest average percentage of total time spent in the US until baseline (61.3%) while Chinese participants had the highest proportion (72.6%).

### Baseline cognitive function

At baseline, Filipino participants had the lowest average ± SD of unstandardized executive function scores (−0.35 ± 0.71), followed by NHPI/other (−0.16 ± 0.76), Chinese (−0.10 ± 0.77) and Japanese (0.02 ± 0.81) participants (Table 1). There were significant differences between AANHPI groups for population-standardized executive function after adjusting for age, gender, education, and cohort (model 1; *F*-value = 10.77, η²*p* = 0.04, *p* < 0.01). Differences persisted after further adjustment for nativity and language (model 2; *F*-value = 11.13, η²*p* = 0.04, *p* < 0.01). In Model 2, the largest difference in executive function was between Filipino and Japanese participants with Filipino participants showing significantly lower baseline EF scores compared with Japanese participants in model 2 (SMD = −0.55; 95% CI −0.79, −0.31; Supplementary Table S2).

For unstandardized verbal episodic memory at baseline, NHPI/Other participants had the lowest average ± SD (−0.18 ± 0.96) followed by Japanese (−0.15 ± 0.97), Chinese (−0.02 ± 1.01), and Filipino (0.003 ± 0.88) participants (Table 1). However, there were no differences between AANPHI groups with regards to population-standardized verbal episodic memory in ANCOVA model 1 (*F*-value = 2.30, η²*p* = 0.008, *p*-value = 0.08) or model 2 (*F*-value = 2.30, η²*p* = 0.008, *p*-value = 0.08). Results from models with multiple imputation were consistent with complete case analyses (Supplementary Table S2).

### Longitudinal cognitive performance

**Fig. 1:**
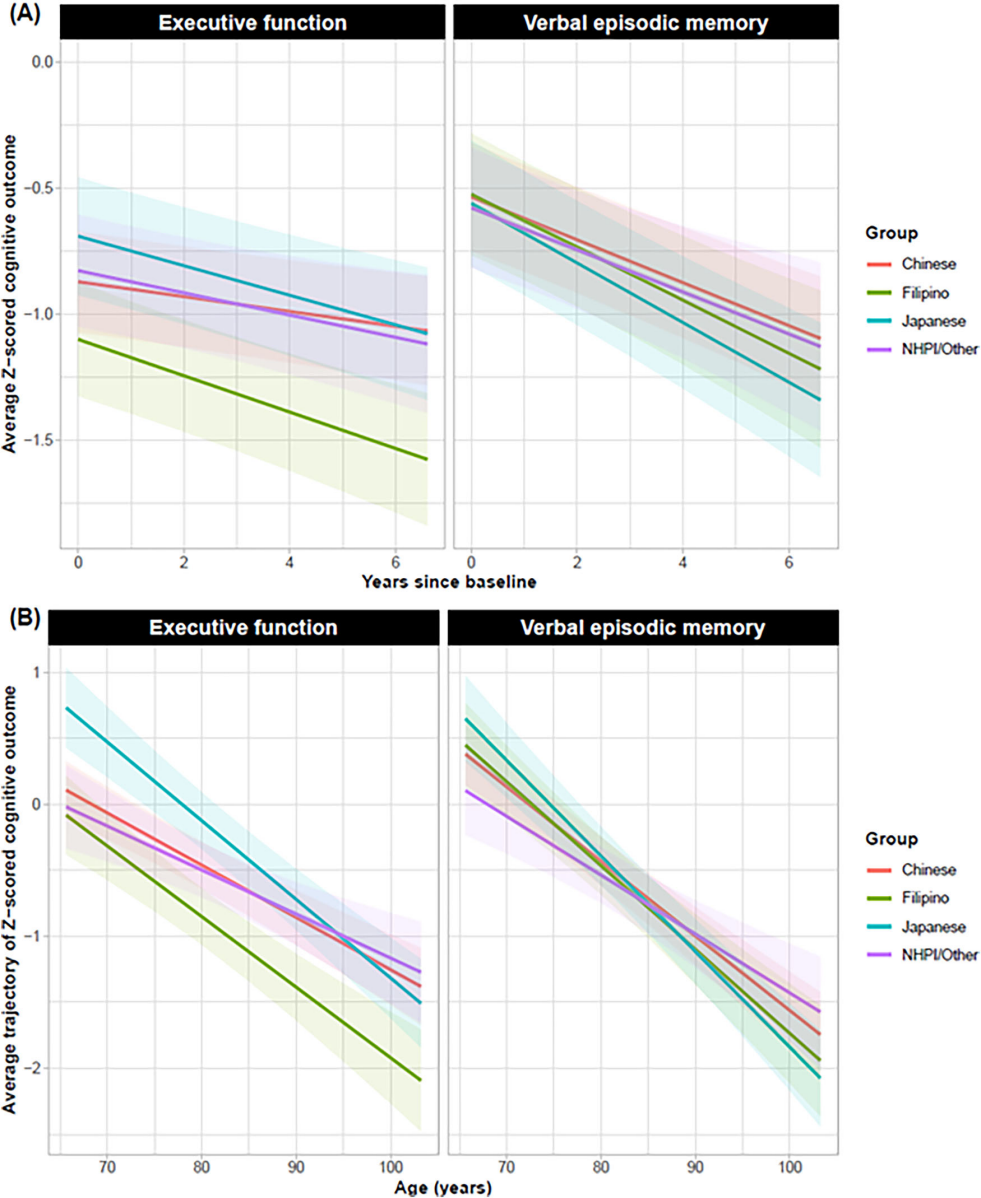
Average trajectories of executive function and verbal episodic memory by Asian American subgroup. **A** Average trajectories of executive function and verbal episodic memory by Asian American subgroup over time. **B** Average trajectories of executive function and verbal episodic memory by Asian American subgroup by age. Trajectories are based on linear mixed-effects models with random intercepts using multiple imputation and adjust for age, gender, education, cohort, interview mode, and practice effects.

The average follow-up time was 2.49 ± 2.18 years. In linear mixed-effects models comparing AANHPI groups, relative to Chinese participants (the largest group), Filipino participants had significantly faster declines in executive function after adjusting for age, gender, education, interview mode, and cohort in model 1 (β = −0.04; 95% CI −0.07, −0.01; Fig. 1; Supplementary Table S3). While not significant, the Japanese and NHPI/Other groups had faster declines in executive function relative to Chinese participants. Results were significant for Japanese participants after further adjustment for nativity and first language in model 2 (β = −0.03; 95% CI -0.06, −0.00). There were no differences in decline for verbal episodic memory. Multiple imputed results were consistent with complete case analyses (Supplementary Table S3) and using age as the timescale (Fig. 1).

### Possible differences by nativity status

**Fig. 2:**
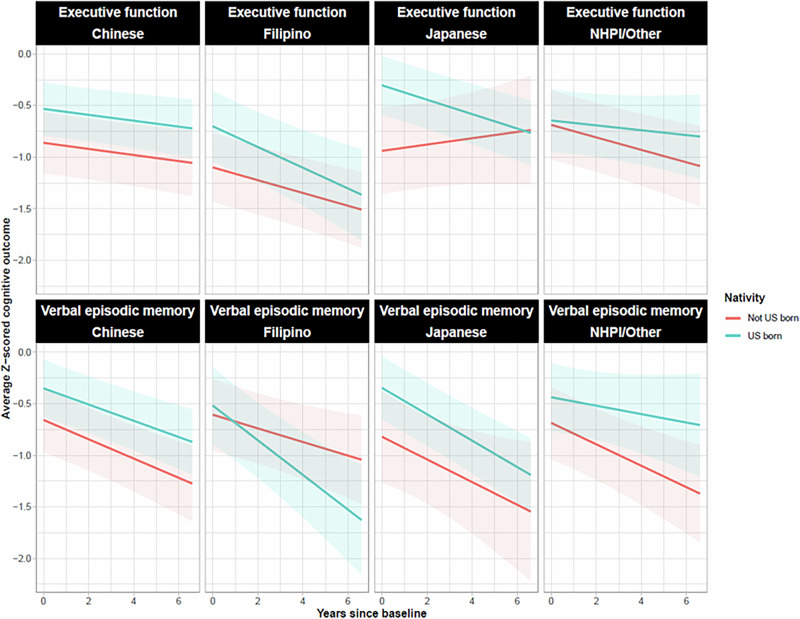
Average trajectories of executive function and verbal episodic memory by Asian American subgroup and nativity over time. Trajectories are based on linear mixed-effects models with random intercepts using multiple imputation and adjust for age, gender, education, cohort, language, interview mode, and practice effects.

At baseline, there were significant differences by group and nativity status for executive function (*p*-interaction = 0.02) but not verbal episodic memory (*p*-interaction = 0.41; Fig. 2). Those who were non-US-born had lower baseline executive function scores across all AANHPI groups compared with those who were born in the US.

For longitudinal change in cognition, there were significant group-by-nativity differences in executive function scores (*p*-interaction = 0.02; Table 2) and verbal episodic memory scores (*p*-interaction = 0.01; Table 3; Fig. 2). In stratified analyses comparing US born and non-US born participants, there were faster executive function declines among US-born Filipino (β_US born_ = −0.07, 95% CI −0.13, −0.02 vs. β_non-US born_ = −0.03, 95% CI −0.07, 0.00) and Japanese participants (β_US born_ = −0.04, 95% CI -0.07, −0.01 vs. β_non-US born_ = 0.06, 95% CI −0.00, 0.12) (Fig. 4). Among the NHPI/Other group, there were faster declines in executive function among the non-US born group and null for the US born group (β_non-__US born_ = −0.03, 95% CI −0.07, 0.01 vs. β_US born_ = 0.00, 95% CI −0.05, 0.06). For verbal episodic memory decline, there was a similar trend such that there were faster declines among US-born Filipino (β_US born_ = −0.09, 95% CI -0.16, −0.01 vs. β_non-US born_ = 0.03, 95% CI −0.03, 0.09) and Japanese participants (β_US born_ = −0.05, 95% CI −0.09, −0.01 vs. β_non-US born_ = −0.02, 95% CI −0.12, 0.09), but slower declines for US born NHPI/Other participants (β_US born_ = 0.04, 95% CI −0.03, 0.11 vs. β_non-US born_ = −0.01, 95% CI −0.08, 0.06).

**Table 2:** Longitudinal associations of Asian American subgroup with executive function over time by select effect modifiers

	Chinese	Filipino	Japanese	NHPI/Other	
Effect modifier	β (95% CI)	β (95% CI)	β (95% CI)	β (95% CI)	*p*-interaction
Nativity					0.02
Non-US Born (*n* = 534)	Reference	−0.03 (−0.07, 0.00)	0.06 (−0.00, 0.12)	−0.03 (−0.07, 0.01)	
US born (*n* = 340)	Reference	−0.07 (−0.13, −0.02)	−0.04 (−0.07, −0.01)	0.00 (−0.05, 0.06)	
First language spoken					0.20
Not English (*n* = 437)	Reference	−0.02 (−0.06, 0.01)	−0.02 (−0.06, 0.01)	−0.04 (−0.09, 0.01)	
English (*n* = 252)	Reference	−0.08 (−0.13, −0.02)	−0.02 (−0.06, 0.03)	0.01 (−0.04, 0.07)	
Education					0.004
≥College (*n* = 517)	Reference	−0.07 (−0.10, −0.03)	−0.04 (−0.07, −0.01)	−0.04 (−0.09, 0.01)	
≤Some college, or tech/trade school (*n* = 356)	Reference	0.01 (−0.04, 0.06)	−0.00 (−0.05, 0.04)	0.02 (−0.02, 0.07)	

Estimates are for group-by-time interactions derived from linear mixed-effect models with random intercepts using multiple imputation. Models adjusted baseline age, gender, practice effects, interview mode, and cohort. Nativity, education, and first language spoken were included in models not tested as a stratification factor. *P*-interaction is derived from likelihood ratio tests.

**Table 3:** Longitudinal associations of Asian American subgroup with verbal episodic memory over time by select effect modifiers

	Chinese	Filipino	Japanese	NHPI/Other	
Effect modifier	β (95% CI)	β (95% CI)	β (95% CI)	β (95% CI)	*p*-interaction
Nativity					0.01
Non-US Born (*n* = 534)	Reference	0.03 (−0.03, 0.09)	−0.02 (−0.12, 0.09)	−0.01 (−0.08, 0.06)	
US born (*n* = 340)	Reference	−0.09 (−0.16, −0.01)	−0.05 (−0.09, −0.01)	0.04 (−0.03, 0.11)	
First language spoken					0.13
Not English (*n* = 437)	Reference	−0.01 (−0.06, 0.05)	−0.05 (−0.11, 0.01)	−0.04 (−0.11, 0.02)	
English (*n* = 252)	Reference	−0.05 (−0.13, 0.04)	−0.04 (−0.10, 0.03)	0.02 (−0.05, 0.10)	
Education					0.18
≥College (*n* = 517)	Reference	−0.04 (−0.10, 0.01)	−0.03 (−0.08, 0.01)	−0.05 (−0.12, 0.02)	
≤Some college, or tech/trade school (*n* = 356)	Reference	0.04 (−0.04, 0.11)	−0.03 (−0.10, 0.04)	0.07 (−0.00, 0.13)	

Estimates are for group-by-time interactions derived from linear mixed-effect models with random intercepts using multiple imputation. Models adjusted baseline age, gender, practice effects, interview mode, and cohort. Nativity, education, and first language spoken were included in models not tested as a stratification factor. *P*-interaction is derived from likelihood ratio tests.

There was a positive correlation of nativity status with first language (*r*_*φ*_ = 0.41; 95% CI 0.34, 0.46; *p* < 0.01). Thus, we repeated models without adjustment for first language. Results were similar for baseline executive function (*p*-interaction < 0.01) and verbal episodic memory (*p*-interaction = 0.41), as well as change in executive function (*p*-interaction = 0.01) and verbal episodic memory (*p*-interaction = 0.01).

### Possible differences by first language spoken

**Fig. 3:**
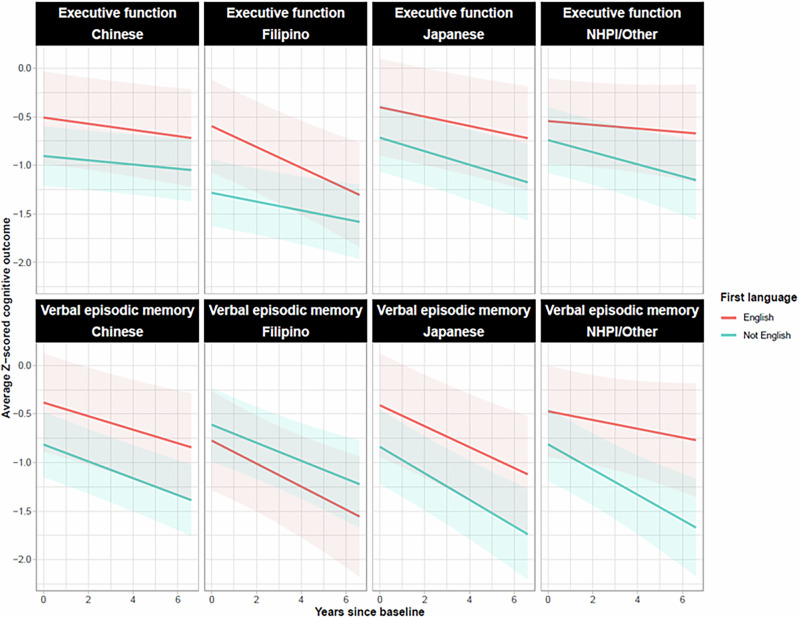
Average trajectories of executive function and verbal episodic memory by Asian American subgroup and first language spoken over time. Trajectories are based on linear mixed-effects models with random intercepts using multiple imputation and adjust for age, gender, education, cohort, nativity, interview mode, and practice effects.

**Fig. 4:**
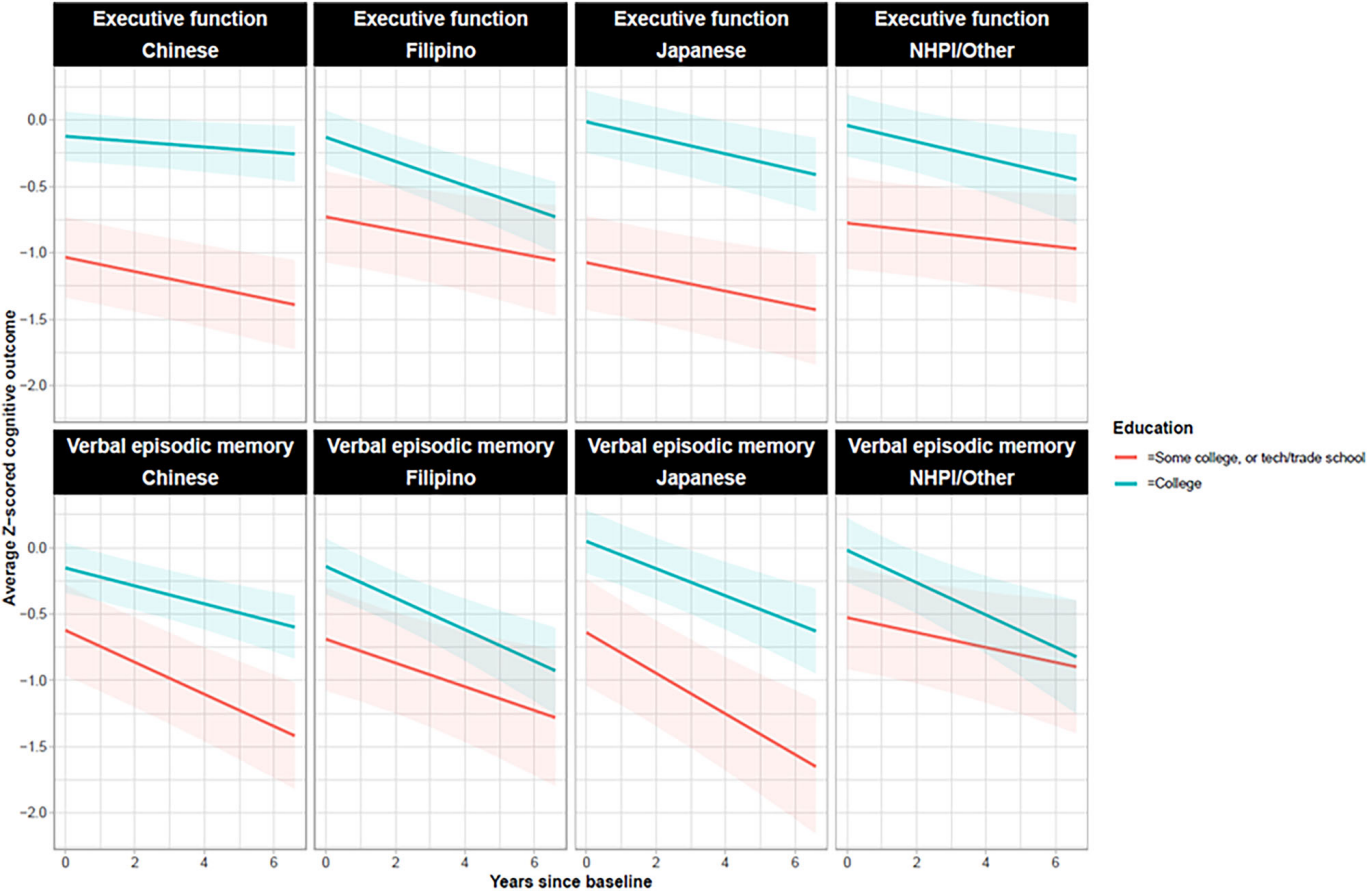
Average trajectories of executive function and verbal episodic memory by Asian American subgroup and educational attainment over time. Trajectories are based on linear mixed-effects models with random intercepts using multiple imputation and adjust for age, gender, education, cohort, nativity, language, interview mode, and practice effects.

There were no significant interactions by first language spoken for baseline executive function (*p*-interaction = 0.23) and verbal episodic memory (*p*-interaction = 0.38; Tables 2 and 3; Fig. 3). There were also no longitudinal differences over time for executive function (*p*-interaction = 0.20) or verbal episodic memory (*p*-interaction = 0.13; Fig. 3). Although interaction terms of group-by-language were not significant, those who did not speak English as their first language generally had lower baseline cognitive function scores while results for cognitive decline were mixed (Fig. 4). We repeated models without adjusting for nativity given the positive correlation of nativity status with first language (*r*_*φ*_ = 0.41; 95% CI 0.34, 0.46; *p* < 0.01); results were similar for baseline and longitudinal models (all *p*’s-interaction > 0.13).

### Possible differences by educational attainment

While baseline interactions were not significant (executive function *p*-interaction = 0.23; verbal episodic memory *p*-interaction = 0.39), those who had attained at least a college education had greater baseline executive function and verbal episodic memory across all AANHPI groups (Fig. 4).

There were group by educational attainment differences over time for executive function (*p*-interaction = 0.004) but not verbal episodic memory (*p*-interaction = 0.18; Fig. 4; Tables 2 and 3). For longitudinal analyses comparing those with ≥college education vs ≤some college or tech/trade school, there were faster declines in executive function for Filipino (β_≥college_ = −0.07, 95% CI −0.10, −0.03 vs. β_≤some college, tech/trade school_ = 0.01, 95% CI −0.04, 0.06) and Japanese participants (β_≥college_ = −0.04, 95% CI −0.07, −0.01 vs. β_≤some college, tech/trade school_ = −0.00, 95% CI −0.05, 0.04). There were weak correlations of education with nativity (*r*_*φ*_ = −0.02; 95% CI −0.09, 0.04; *p* = 0.49) and first language (*r*_*φ*_ = −0.03; 95% CI −0.10, 0.05; *p* = 0.49).

### Sensitivity analysis: possible differences by cohort

After stratifying by cohort, baseline executive function differences between AANHPI groups remained in fully adjusted models (KHANDLE: *F*-value = 18.08, η²*p* = 0.10, *p* < 0.01; *LifeAfter90*: *F*-value = 6.47, η²*p* = 0.05, *p* < 0.01). There were no differences in baseline verbal episodic memory for stratified analyses within KHANDLE (*F*-value = 1.53, η²*p* = 0.01, *p* = 0.20) and *LifeAfter90* (*F*-value = 0.68, η²*p* = 0.01, *p* = 0.56), consistent with our primary pooled analysis.

In longitudinal cohort-stratified analyses, there were differences in cognitive decline for *LifeAfter90* but not KHANDLE participants. Specifically, there were faster declines in executive function for Filipino (β = −0.11; 95% CI −0.18, −0.05; Supplementary Table S4) and Japanese (β = −0.07; 95% CI −0.11, −0.03) *LifeAfter90* participants relative to Chinese *LifeAfter90* participants. There were faster declines in verbal episodic memory for Japanese (β = −0.10; 95% CI −0.16, −0.05) relative to Chinese *LifeAfter90* participants.

## Discussion

In this study of 875 diverse AANHPI in Northern California, we found significant baseline and longitudinal differences in executive function between disaggregated subgroups. These associations were more pronounced in the *LifeAfter90* cohort. Nativity, but not first language spoken, moderated these associations such that US-born participants had higher baseline executive function but faster decline. Educational attainment only moderated longitudinal associations with executive function. Overall, our findings suggest potential differences in domain specific cognitive function across AANHPI subgroups, supporting the value of data disaggregation for this ethnically heterogeneous group.

In our baseline analyses, there were differences in executive function, but not verbal episodic memory, by ethnic subgroup. Posthoc tests revealed the largest differences between Filipino and Japanese participants: Filipino participants exhibited lower baseline executive function scores when compared with Japanese participants. Chinese participants had greater baseline executive function scores compared with Filipino participants but lower compared with Japanese participants. In longitudinal analyses, Filipino and Japanese participants had faster declines in executive function compared with Chinese participants. In sensitivity analyses, associations with decline were most apparent in the *LifeAfter90* but not KHANDLE cohort, suggesting age- and cohort-related differences at play. Nonetheless, these findings align with a KPNC electronic medical records-based study which found that Filipino and Japanese participants had a 20% and 8%, respectively, greater incidence of all-cause dementia compared with Chinese participants^3^.

Differences in executive function, particularly among Filipino and Japanese American participants, may have emerged due to several factors that we did not study here^3,4,9^ including differences in cardiometabolic factors such as diabetes^29–32^, hypertension^33^ and dyslipidemia^34^, which are established dementia risk factors^35–37^. For example, Filipino Americans have higher prevalence of hypertension compared with other groups^33^, which is associated with greater dementia risk^35^. Our null findings may be explained by heterogeneity in dementia risk factors, rather than potential differences in language proficiency given the large language component in both executive function and verbal episodic memory assessments^38,39^. Higher powered studies are needed to test cardiometabolic-related differences across multiple subgroups, as simple adjustment results in overadjustment if they are intermediates^40^.

In effect modification analyses, those who were born in the US or had at least a college education had greater baseline executive function scores. This aligns with prior work among Hispanic/Latino^13,14^, Korean^15^, Japanese^21^, and Chinese American^20,41^ study populations in the US which suggests that greater acculturation is associated with better cognitive function. This is also consistent with a KPNC study which found that greater educational attainment was associated with lower dementia incidence across different AANHPI groups^6^. A recent study of 158 Chinese, Korean, and Vietnamese American participants in Southern California found that greater acculturation, measured by the Suinn-Lew Asian Self-Identify Acculturation scale, was associated with better global cognition measured by the Montreal Cognitive Assessment (MoCA)^19^, which aligns with our findings. However, they did not test between-AANHPI group differences. Our work extends prior evidence by examining cognitive domain-specific associations across disaggregated subgroups.

Interestingly, we found that Japanese and Filipino participants who were born in the US and had ≥college education had faster declines in executive function and verbal episodic memory. This could partly be explained by the Immigrant Paradox whereby those not born in the US have better health outcomes^42^, driven by US immigration policy imposing a selection bias favoring Asian immigrants with higher education and social standing^26^. Certain AANHPI groups may have different levels of cognitive reserve, perhaps reflective of different lifecourse and environmental exposures, that allows for greater preservation of cognitive function up to a certain point, then decline at a faster rate.

Contrary to our hypothesis, we did not find differences across AANHPI groups in baseline and longitudinal cognitive function by whether English was their first language spoken or not. While our study did not directly assess bilingualism or multilingualism, it may play a role in cognitive reserve^43,44^. In other work, those who are bilingual, versus not, had lower performance on verbal fluency tasks based on timed assessments, potentially due to linguistic interference that may impact category fluency^45^. Others have found that bilingual participants perform greater at executive function tasks^46^.

Our study has multiple limitations. First, KHANDLE and *LifeAfter90* included AANHPI participants who identified predominantly as Chinese, Japanese, and Filipino, and all resided in Northern California. This limits generalizability to AANHPI groups in this region. Notably, a larger proportion of our participants identified as Chinese American compared with other AANHPI subgroups. Further work and replication are needed across many AANHPI subgroups in different areas of the US. Second, since participants were initially screened for English or Spanish proficiency and study visits were conducted in either language, our sample is restricted to AANHPI who speak either of those languages and may be inherently more acculturated at study onset. Additionally, we were unable to adjust for English or Spanish proficiency, which may impact performance on cognitive assessments conducted in non-primary language. Third, we were limited to studying culture-related proxies based on nativity, language learned, and education, which may not fully capture the experience of acculturation. Other studies examined this dimension on other facets such as American media consumption^20^ or scale-based assessments^19^. We were also unable to include additional confounders, such as occupational complexity and continuous operationalization of education, that may impact cognition and partly explain differences^47^. Because our cognitive outcomes were composites of multiple tests, future work should further probe on these specific outcomes (e.g., category and letter fluency). Additionally, there may be selection bias in that all participants had access to healthcare as long-term KPNC members, thus limiting generalizability to AANHPI who were 82 years old on average and with healthcare access^48^, but inherently reduces confounding due to healthcare access in relation to cognitive differences. Lastly, we had brief follow-up of 2.49 years. Because existing literature on cognitive function in AANHPI focus on single groups and are largely cross-sectional^15,19–21^, with few examining cognitive change^41,49^, future research should investigate potential differences in cognitive function over a longer timeframe.

This study has several strengths. KHANDLE and *LifeAfter90* included diverse participants, spanning multiple AANHPI subgroups who represent an understudied population in cognitive aging research^7–11^. Prior AANHPI work is primarily limited to electronic medical record-based dementia diagnoses^3,5,6,11,33,50,51^. We improve upon this by leveraging rich assessment data on domain specific cognitive function and sociodemographic characteristics, such as nativity and first language spoken, that are typically absent in electronic medical record studies. Lastly, we were able to minimize the impact of selection bias due to missing data with multiple imputation^52^.

In this study of 875 AANHPI adults residing in Northern California, we found differences in cognitive function across AANHPI subgroups. Specifically, our results suggest potential differences in baseline executive function that persisted with time. These findings differed by nativity and education, and were more pronounced in those over age 90. Our findings must be caveated in light of several limitations including the brief follow-up and limited generalizability of this highly selected cohort. Nonetheless, this is the first study to our knowledge to assess these differences and moderating factors across multiple AANHPI subgroups. Further work examining the mechanisms of AANHPI disaggregated subgroup differences is crucial given that they are among the fastest growing populations in the US^53^, with increasing ADRD burden. These data are necessary to continue to understand and reduce disparities in cognitive function between AANHPI subgroups.

## Methods

### Study population

This study included data from two ongoing prospective cohort studies: Kaiser Healthy Aging and Diverse Life Experiences (KHANDLE) and *LifeAfter90*^17,47,48,54,55^. The KHANDLE and *LifeAfter90* studies were harmonized given overlap in the cognitive measures and geographic region of participants. KHANDLE began in April 2017 and *LifeAfter90* began in July 2018, and enrollment is ongoing. The goal of both studies is to assess lifecourse factors in relation to cognitive and brain aging. Both studies included participants who are long-term members of Kaiser Permanente Northern California (KPNC), resided in the San Francisco Bay and Sacramento areas of California, spoke English or Spanish, were at least 65 years old for KHANDLE or 90 years old for *LifeAfter90*, and, for KHANDLE participants, had participated in at least one of the Kaiser Permanente Multiphasic Health Checkups (MHC) between 1964 and 1996. Participants were recruited using a stratified random sampling approach, yielding approximately similar proportions of participants across backgrounds and different levels of education. Potential participants self-rated their language preferences and English and Spanish proficiency during screening calls. Those who self-reported not speaking English or Spanish well or at all were not invited to participate in KHANDLE and LifeAfter90. Interviewers also rated the potential participant’s communication during screening to determine if it was feasible for them to participate in English or Spanish. At baseline, all participants were free of a diagnosis of dementia, hospice care, or dialysis in their medical record. KHANDLE participants were interviewed every 16–18 months and *LifeAfter90* participants were interviewed every 6 months.

All participants provided informed consent and study protocols were approved by the KPNC and University of California, Davis Institutional Review Boards (#1279409 and #1278966). Research was performed in accordance with the Declaration of Helsinki. The present study followed the Strengthening the Reporting of Observational Studies in Epidemiology (STROBE) reporting guidelines for cohort studies^56^.

For this study, we included 901 participants from KHANDLE and *LifeAfter90* who self-identified as at least one AANHPI ethnic group (defined below). Of the 3649 participants in KHANDLE and *LifeAfter90*, we included 901. Of those, we excluded 26 with limited demographic data and missing data on executive function and verbal episodic memory at baseline (retaining 4 missing verbal memory but not executive function), yielding a final analytic sample of 875 participants. This analysis considered up to 4 waves of data for KHANDLE participants and 8 waves of data for *LifeAfter90* participants.

### Measures

Participants self-reported their ethnicity via a questionnaire. This study included AANHPI participants who self-identified as Chinese, Japanese, Korean, Filipino, Other Southeast Asian (e.g., Cambodian, Laotian), Native Hawaiian, Samoan, Other Pacific Islander, or Other Asian ethnic group. Participants who reported multiple ethnicities were categorized into “Other.” Due to limited sample sizes of certain AANHPI ethnic groups, we categorized participants into 4 groups: (1) Chinese, (2) Filipino, (3) Japanese, and (4) Native Hawaiian, Pacific Islander, multiple reported ethnicity, or other Asian ethnic group (NHPI/Other). We acknowledge that there are ethnically and culturally distinct groups in the NHPI/Other subgroup, but grouped them due to limited sample size for subsequent statistical analyses; we report their distribution in Supplementary Table 1.

Cognitive function was assessed via the psychometrically validated Spanish and English Neuropsychological Scales (SENAS) at each study visit^38,39^. There were composite indices for two cognitive domains of interest: (1) verbal episodic memory, and (2) executive function. Verbal episodic memory scores were derived from multi-trial word list learning tasks^38^. Executive function scores were derived from components of category fluency, phonemic fluency, and working memory (via digit- and visual span backward, list sorting)^39^. SENAS administration was modified in *LifeAfter90* using shortened versions of multiple tasks^54^. We used z-scored SENAS outcomes using mean and standard deviation (SD) values at Visit 1 for the full analytic sample (see Table 1).

We included age at baseline (years), gender (women; men), education level (≤High school/GED; some college or tech/trade school; college; graduate school), nativity (US born; non-US born), first language spoken (Not English; English) and cohort (*LifeAfter90*; KHANDLE), as covariates of interest. Education was treated as a categorical variable, rather than continuous, because associations between education and different health outcomes are typically non-monotonic^57,58^. We considered nativity and first language spoken as culture-related effect modifiers^13,14,16,22^. For those who were not born in the US, we also calculated the percentage of life years spent in the US (i.e., Age at baseline - age at immigration to US/age at baseline)^29^. Due to the COVID-19 pandemic, some assessments were conducted via phone, and thus we included a covariate for interview mode (phone; in-person). To account for practice effects in longitudinal models, we included a visit 1 indicator (yes; no)^59,60^.

### Statistical analysis

We described participant characteristics by AANHPI group using mean (SD) or median (minimum, maximum) for continuous variables, and counts (percentages) for categorical variables.

To compare baseline differences in memory and executive function across ethnic groups, we first described unstandardized scores using means and SD. We then tested differences across subgroups using analysis of covariance (ANCOVA) tests, adjusted for age, gender, education, interview mode, and cohort in model 1. Model 2 additionally adjusted for nativity and first language spoken. *F*-tests and partial eta-squared (η²p) are reported as effect sizes. In posthoc tests, we calculated average population standardized mean differences (SMD) in baseline cognitive function to quantify differences between AANHPI groups.

To assess longitudinal differences in memory and executive change across subgroups, we fit successively adjusted linear mixed-effects models with random intercepts, using years since baseline as the timescale^61^. Our primary model, Model 1, included AANHPI group, time (years since baseline), and a group-by-time interaction term, adjusted for baseline age, gender, education, cohort, interview mode, and practice effects. Model 2 further adjusted for nativity and first language spoken, which we considered culture-related moderators. Linear mixed-effects models did not include random slopes due to limited model convergence. For estimates of group-by-time interactions, the Chinese participant group was selected as the reference group because it was the largest. For visualization, we plotted average trajectories of executive function and memory over time by AANHPI subgroup.

In effect modification analyses, we examined whether cognitive function and change differed by nativity status. Likelihood ratio or Wald tests were used to compare model 2 with and without interactions. To compare modifier-level estimates, we then stratified models by nativity status, comparing each cognitive outcome across AANHPI group and nativity status. This procedure was repeated to test first language spoken and education as effect measure modifiers. We defined educational attainment dichotomously as “≤some college, tech/trade school” vs. “≥college” due to limited sample size^6^. The Phi coefficient was used to estimate correlations between nativity, education, and first language spoken out of concern for potential multicollinearity.

To handle missing data on baseline education (0.2% missing) and first language data (21.3% missing), we used multiple imputation by chained equations with 100 imputations and 5 iterations^62^. We reported pooled estimates using Rubin’s rules, accounting for between- and within-imputation variance^52^. We performed several sensitivity analyses. First, we presented complete case analysis results for our baseline and longitudinal models. Second, age-based longitudinal analyses were tested^61^. Lastly, we stratified by cohort given differences in age and differential follow-up intervals between the KHANDLE and *LifeAfter90* studies.

All statistical analyses were conducted using R version 4.4.0 (R Core Team 2024). Our main objective was to describe memory and executive function abilities and decline across AANHPI groups who have been understudied in this area of research. Accordingly, we did not control for multiple comparisons out of concern for reducing type I error in exchange for increasing type II error^63^.

## Supplementary information

Supplementary Information

## Data Availability

Data are available upon approved request at https://sites.google.com/g.ucla.edu/khandle-study-site/home.

## References

[1] Krogstad, J. M. & Im, C. Key facts about Asians in the U.S. Pew Research Center. https://www.pewresearch.org/short-reads/2025/05/01/key-facts-about-asians-in-the-us/ (2025).

[2] United States Census Bureau. 2023 National Population Projections Tables: Main Series. United States Census Bureau. https://www.census.gov/data/tables/2023/demo/popproj/2023-summary-tables.html (2025).

[3] Mayeda, E. R., Glymour, M. M., Quesenberry, C. P. J. & Whitmer, R. A. Heterogeneity in 14-year Dementia Incidence Between Asian American Subgroups. *Alzheimer Dis. Assoc. Disord.* **31**, 181 (2017). 28406845 10.1097/WAD.0000000000000189 PMC5568954

[4] Matthews, K. A. et al. Racial and ethnic estimates of Alzheimer’s disease and related dementias in the United States (2015–2060) in adults aged ≥65 years. *Alzheimers Dement.* **15**, 17–24 (2019). 30243772 10.1016/j.jalz.2018.06.3063 PMC6333531

[5] Rojas-Saunero, L. P. et al. Sex/gender differences in lifetime dementia risk among Asian American and White older adults. *NPJ Dement* **1**, 32 (2025). 41098619 10.1038/s44400-025-00038-8 PMC12518132

[6] Hayes-Larson, E. et al. Association of Education With Dementia Incidence Stratified by Ethnicity and Nativity in a Cohort of Older Asian American Individuals. *JAMA Netw. Open* **6**, e231661 (2023). 36877520 10.1001/jamanetworkopen.2023.1661 PMC9989900

[7] Ðoàn, L. N. et al. Turning the Health Equity Lens to Diversity in Asian American Health Profiles. *Annu. Rev. Public Health* **45**, 169–193 (2024). 38134402 10.1146/annurev-publhealth-060222-023852

[8] Kanaya, A. M. et al. Knowledge Gaps, Challenges, and Opportunities in Health and Prevention Research for Asian Americans, Native Hawaiians, and Pacific Islanders: A Report From the 2021 National Institutes of Health Workshop. *Ann. Intern. Med.* **175**, 574–589 (2022). 34978851 10.7326/M21-3729 PMC9018596

[9] Lim, S. et al. Alzheimer’s Disease and its Related Dementias among Asian Americans, Native Hawaiians, and Pacific Islanders: A Scoping Review. *J. Alzheimers Dis.* **77**, 523–537 (2020). 32675416 10.3233/JAD-200509 PMC8638681

[10] Holland, A. T. & Palaniappan, L. P. Problems with the collection and interpretation of Asian-American health data: omission, aggregation, and extrapolation. *Ann. Epidemiol.* **22**, 397–405 (2012). 22625997 10.1016/j.annepidem.2012.04.001 PMC4324759

[11] Zhu, Y. et al. A systematic review/meta-analysis of prevalence and incidence rates illustrates systemic underrepresentation of individuals racialized as Asian and/or Asian-American in ADRD research. *Alzheimers Dement.* **20**, 4315–4330 (2024). 38708587 10.1002/alz.13820 PMC11180860

[12] Adkins-Jackson, P. B., et al. The structural and social determinants of Alzheimer’s disease related dementias. *Alzheimers Dement*. 10.1002/alz.13027 (2023). 10.1002/alz.13027 PMC10599200 37074203

[13] Mendoza, L. et al. The effect of acculturation on cognitive performance among older Hispanics in the United States. *Appl. Neuropsychol. Adult* **29**, 163–171 (2022). 32116033 10.1080/23279095.2020.1725888 PMC12108945

[14] Martinez-Miller, E. E. et al. Longitudinal Associations of US Acculturation With Cognitive Performance, Cognitive Impairment, and Dementia. *Am. J. Epidemiol.* **189**, 1292–1305 (2020). 32440686 10.1093/aje/kwaa088 PMC7604518

[15] Choi, E. Y., Jang, Y. & Chiriboga, D. A. Gender as a Moderator of the Effect of Education and Acculturation on Cognitive Function: A Study of Older Korean Immigrants. *J. Aging Health* **32**, 1659–1666 (2020). 32783692 10.1177/0898264320950554 PMC12447837

[16] Alam, R. B. et al. Is Acculturation Associated with the Cognitive Performance of Older Hispanics. *J. Alzheimers Dis.* **85**, 535–544 (2022). 34842186 10.3233/JAD-210502

[17] Meyer, O. L., et al. Generation and Age of Immigration on Later Life Cognitive Performance in KHANDLE. *Int. Psychogeriatr*. 1–12, 10.1017/S1041610220003774 (2020). 10.1017/S1041610220003774 PMC8219806 33353575

[18] Lamar, M. et al. Acculturation in Context: The Relationship Between Acculturation and Socioenvironmental Factors With Level of and Change in Cognition in Older Latinos. *J. Gerontol. B Psychol. Sci. Soc. Sci.* **76**, e129–e139 (2021). 32918471 10.1093/geronb/gbaa156 PMC7955974

[19] Jang, Y., Kawachi, I. & Lee, S. Education, Acculturation, and Network Diversity as Promoters of Cognitive Function: A Study of Older Chinese, Korean, and Vietnamese Americans in Southern California. *J. Aging Health*. 10.1177/08982643251370411 (2025). 10.1177/08982643251370411 40833152

[20] Li, M. et al. Family type and cognitive function in older Chinese Americans: acculturation as a moderator. *Aging Ment. Health* **26**, 1642–1653 (2022). 34038643 10.1080/13607863.2021.1926426 PMC8718330

[21] Enobi, Y., Kemmotsu, N., Robinson, E. & Murphy, C. Effects of language and acculturation on neurocognitive performance of Japanese Americans. *Neuropsychology* **36**, 651–663 (2022). 35951411 10.1037/neu0000839 PMC9805380

[22] Meza, E. et al. Perceived Discrimination, Nativity, and Cognitive Performance in a Multiethnic Study of Older Adults: Findings From the Kaiser Healthy Aging and Diverse Life Experiences Study. *J. Gerontol. A Biol. Sci. Med. Sci.* **77**, e65–e73 (2021). 10.1093/gerona/glab170 PMC8824601 34125189

[23] Walton, J. & Truong, M. A review of the model minority myth: Understanding the social, educational and health impacts. *Ethn. Racial Stud.* **46**, 391–419 (2023).

[24] Kim, J. H. J., Lu, Q. & Stanton, A. L. Overcoming constraints of the model minority stereotype to advance Asian American health. *Am. Psychologist* **76**, 611–626 (2021). 10.1037/amp0000799 PMC8384115 34410738

[25] Goh, J. X., Lei, R. F. & Zou, L. X. Positioning Asian Americans in social cognition. *Soc. Personal. Psychol. Compass* **17**, e12760 (2023).

[26] Junn, J. From Coolie to Model Minority. *Du Bois Rev.* **4**, 355–373 (2007).

[27] Morley, J. E. et al. Brain health: the importance of recognizing cognitive impairment: an IAGG consensus conference. *J. Am. Med. Dir. Assoc.* **16**, 731–739 (2015). 26315321 10.1016/j.jamda.2015.06.017 PMC4822500

[28] Bondi, M. W. et al. Neuropsychological Contributions to the Early Identification of Alzheimer’s Disease. *Neuropsychol. Rev.* **18**, 73–90 (2008). 18347989 10.1007/s11065-008-9054-1 PMC2882236

[29] Araneta, M. R. Engaging the ASEAN Diaspora: Type 2 Diabetes Prevalence, Pathophysiology, and Unique Risk Factors among Filipino Migrants in the United States. *J. ASEAN Fed. Endocr. Soc.* **34**, 126–133 (2019). 33442147 10.15605/jafes.034.02.02 PMC7784106

[30] Araneta, M. R. G., Grandinetti, A. & Chang, H. K. A1C and Diabetes Diagnosis Among Filipino Americans, Japanese Americans, and Native Hawaiians. *Diab Care* **33**, 2626–2628 (2010). 10.2337/dc10-0958 PMC2992202 20833866

[31] Araneta, M. R. G., Wingard, D. L. & Barrett-Connor, E. Type 2 Diabetes and Metabolic Syndrome in Filipina-American Women: a high-risk nonobese population. *Diab Care* **25**, 494–499 (2002). 10.2337/diacare.25.3.494 11874936

[32] Lee, J. W. R., Brancati, F. L. & Yeh, H. C. Trends in the Prevalence of Type 2 Diabetes in Asians Versus Whites: Results from the United States National Health Interview Survey, 1997–2008. *Diab Care* **34**, 353–357 (2011). 10.2337/dc10-0746 PMC3024348 21216863

[33] Gordon, N. P., Lien, I. C., Rana, J. S. & Lo, J. C. U. S. Filipino Adults Have Elevated Prevalence of Hypertension Across the Adult Lifespan: Findings From a Cross-Sectional Electronic Health Record Study. *AJPM Focus* **3**, 100211 (2024). 38633726 10.1016/j.focus.2024.100211 PMC11021886

[34] Frank, A. T. H. et al. Racial/ethnic differences in dyslipidemia patterns. *Circulation* **129**, 570–579 (2014). 24192801 10.1161/CIRCULATIONAHA.113.005757 PMC4212818

[35] Livingston, G., et al. Dementia prevention, intervention, and care: 2024 report of the Lancet standing Commission. *Lancet*, 10.1016/S0140-6736(24)01296-0 (2024). 10.1016/S0140-6736(24)01296-0 39096926

[36] Dove A., et al. Cardiometabolic multimorbidity accelerates cognitive decline and dementia progression. *Alzheimers Dement.*, 10.1002/alz.12708 (2022). 10.1002/alz.12708 35708183

[37] Dove, A. et al. Cardiometabolic disease, cognitive decline, and brain structure in middle and older age. *Alzheimers Dement.* **16**, e12566 (2024). 10.1002/dad2.12566 PMC11002777 38595913

[38] Mungas, D., Reed, B. R., Crane, P. K., Haan, M. N. & González, H. Spanish and English Neuropsychological Assessment Scales (SENAS): Further Development and Psychometric Characteristics. *Psychol. Assess.* **16**, 347–359 (2004). 15584794 10.1037/1040-3590.16.4.347

[39] Crane, P. K. et al. Composite scores for executive function items: Demographic heterogeneity and relationships with quantitative magnetic resonance imaging. *J. Int. Neuropsychol. Soc.* **14**, 746–759 (2008). 18764970 10.1017/S1355617708081162 PMC2683684

[40] Schisterman, E. F., Cole, S. R. & Platt, R. W. Overadjustment bias and unnecessary adjustment in epidemiologic studies. *Epidemiology* **20**, 488–495 (2009). 19525685 10.1097/EDE.0b013e3181a819a1 PMC2744485

[41] Tang, F., Li, K., Rauktis, M. E., Buckley, T. D. & Chi, I. Immigration Experience and Cognitive Function Trajectories Among Older Chinese Immigrants. *J. Gerontol. B Psychol. Sci. Soc. Sci.* **78**, 124–135 (2023). 35988160 10.1093/geronb/gbac120 PMC9890920

[42] Teruya, S. A. & Bazargan-Hejazi, S. The Immigrant and Hispanic Paradoxes: A Systematic Review of Their Predictions and Effects. *Hisp. J. Behav. Sci.* **35**, 486–509 (2013). 26120244 10.1177/0739986313499004 PMC4478591

[43] Craik, F. I. M., Bialystok, E. & Freedman, M. Delaying the onset of Alzheimer disease: bilingualism as a form of cognitive reserve. *Neurology* **75**, 1726–1729 (2010). 21060095 10.1212/WNL.0b013e3181fc2a1c PMC3033609

[44] Zahodne, L. B., Schofield, P. W., Farrell, M. T., Stern, Y. & Manly, J. J. Bilingualism does not alter cognitive decline or dementia risk among Spanish-speaking immigrants. *Neuropsychology* **28**, 238–246 (2014). 24188113 10.1037/neu0000014 PMC3947427

[45] Luo, L., Luk, G. & Bialystok, E. Effect of language proficiency and executive control on verbal fluency performance in bilinguals. *Cognition* **114**, 29–41 (2010). 19793584 10.1016/j.cognition.2009.08.014

[46] Grundy, J. G. The effects of bilingualism on executive functions: an updated quantitative analysis. *J. Cult. Cogn. Sci.* **4**, 177–199 (2020).

[47] Soh, Y. et al. Association of primary lifetime occupational cognitive complexity and cognitive decline in a diverse cohort: Results from the KHANDLE study. *Alzheimers Dement* **19**, 3926–3935 (2023). 37057753 10.1002/alz.13038 PMC10517075

[48] Hayes-Larson, E. et al. Accounting for lack of representation in dementia research: Generalizing KHANDLE study findings on the prevalence of cognitive impairment to the California older population. *Alzheimers. Dement.* **18**, 2209–2217 (2022). 35102726 10.1002/alz.12522 PMC9339583

[49] Kallianpur, K. J. et al. Cross-sectional and longitudinal associations between late-life depressive symptoms and cognitive deficits: 20-year follow-up of the Kuakini Honolulu-Asia Aging Study. *Arch. Gerontol. Geriatr.* **127**, 105551 (2024). 38968756 10.1016/j.archger.2024.105551 PMC11401759

[50] Hayes-Larson, E. et al. Heterogeneity in the effect of type 2 diabetes on dementia incidence in a diverse cohort of Asian American and non-Latino White older adults. *Am. J. Epidemiol.* **193**, 1261–1270 (2024). 38949483 10.1093/aje/kwae051 PMC11369220

[51] Mobley, T. M. et al. Neighborhood disadvantage and dementia incidence in a cohort of Asian American and non-Latino White older adults in Northern California. *Alzheimers. Dement.* **19**, 296–306 (2023). 35388625 10.1002/alz.12660 PMC9535033

[52] Little R. J. A., Rubin D. B. *Statistical Analysis with Missing Data*, 3rd ed. (John Wiley & Sons; 2019).

[53] Budiman A., Ruiz N. G. Key facts about Asian Americans, a diverse and growing population. Pew Research Center. https://www.pewresearch.org/short-reads/2021/04/29/key-facts-about-asian-americans/ (2021).

[54] Lam J. O., et al. Gender differences in the association between education and late-life cognitive function in the LifeAfter90 Study: A multiethnic cohort of the oldest-old. *Alzheimers Dement*. 10.1002/alz.14217 (2024). 10.1002/alz.14217 PMC12060128 39254234

[55] Posis, A. I. B. et al. Depressive symptoms are associated with hippocampal volume in the oldest-old: the LifeAfter90 study. *Psychiatry Res. Neuroimag.* **348**, 111967 (2025). 10.1016/j.pscychresns.2025.111967 PMC11908882 39999633

[56] von Elm, E. et al. The Strengthening the Reporting of Observational Studies in Epidemiology (STROBE) Statement: guidelines for reporting observational studies. *Int. J. Surg.* **12**, 1495–1499 (2014). 25046131 10.1016/j.ijsu.2014.07.013

[57] Sharp, E. S. & Gatz, M. The Relationship between Education and Dementia An Updated Systematic Review. *Alzheimer Dis. Assoc. Disord.* **25**, 289–304 (2011). 21750453 10.1097/WAD.0b013e318211c83c PMC3193875

[58] Everett, B. G., Rehkopf, D. H. & Rogers, R. G. The Nonlinear Relationship between Education and Mortality: An Examination of Cohort, Race/Ethnic, and Gender Differences. *Popul. Res. Policy Rev.* **32**, 10.1007/s11113-013-9299-0 (2013). 10.1007/s11113-013-9299-0 PMC3839428 24288422

[59] Chen, R. et al. Pragmatic approaches to handling practice effects in longitudinal cognitive aging research. *Alzheimers. Dement.* **19**, 4028–4036 (2023). 37199336 10.1002/alz.13067 PMC10524983

[60] Vivot, A. et al. Jump, Hop, or Skip: Modeling Practice Effects in Studies of Determinants of Cognitive Change in Older Adults. *Am. J. Epidemiol.* **183**, 302–314 (2016). 26825924 10.1093/aje/kwv212 PMC4753282

[61] Hayes-Larson, E. et al. Approaches to Timescale Choice in Cognitive Aging Research and Potential Implications for Estimated Exposure Effects: Coordinated Analyses in 10 Cohorts of Older Adults. *Epidemiology* **36**, 560–571 (2025). 40164562 10.1097/EDE.0000000000001859 PMC12211680

[62] van Buuren, S. & Groothuis-Oudshoorn, K. mice: multivariate imputation by chained equations in R. *J. Stat. Softw.* **45**, 1–67 (2011).

[63] Rothman, K. J. Six persistent research misconceptions. *J. Gen. Intern. Med.* **29**, 1060–1064 (2014). 24452418 10.1007/s11606-013-2755-z PMC4061362

