# Supplementary material for: Cognitive performance among diverse Asian American subgroups: exploring the role of nativity, language, and education
Source: NPJ Dement. 2026 Jul 3;2(1):51. doi: 10.1038/s44400-026-00108-5 (PMC13331744; doi:10.1038/s44400-026-00108-5)
Supplement: Supplementary file 1 — Supplementary Information [file 44400_2026_108_MOESM1_ESM.pdf]

Supplementary Material

Table S1: Disaggregated description of Native Hawaiian, Pacific Islander, multiple reported ethnicity, or other Asian ethnic group (NHPI/Other)

Ethnicity	n (%)
South Asian (Indian, Pakistani, etc.)	42 (32%)
Korean	6 (5%)
Other Southeast Asian (Cambodian, Laotian, etc.)	8 (6%)
Native Hawaiian, Samoan, Other Pacific Islander	7 (5%)
Multiple ethnicities reported	68 (52%)

Table S2: Standardized mean differences (SMD) in baseline executive function or verbal episodic memory between Asian American subgroups

	Executive function		Verbal episodic memory	
	Model 1	Model 2	Model 1	Model 2
Group contrast	SMD (95%)	SMD (95%)	SMD (95%)	SMD (95%)
Multiple imputation analysis				
Chinese vs. Filipino	0.49 (0.30, 0.69)	0.31 (0.11, 0.52)	0.05 (-0.14, 0.24)	0.06 (-0.14, 0.27)
Chinese vs. Japanese	-0.36 (-0.54, -0.19)	-0.24 (-0.42, -0.06)	0.04 (-0.14, 0.21)	0.03 (-0.15, 0.21)
Chinese vs. NHPI/Other	-0.06 (-0.25, 0.14)	-0.10 (-0.31, 0.10)	0.09 (-0.11, 0.28)	0.09 (-0.11, 0.29)
Filipino vs. Japanese	-0.86 (-1.08, -0.64)	-0.55 (-0.79, -0.31)	-0.01 (-0.23, 0.21)	-0.03 (-0.27, 0.21)
Filipino vs. NHPI/Other	-0.55 (-0.79, -0.31)	-0.42 (-0.66, -0.17)	0.04 (-0.21, 0.28)	0.03 (-0.22, 0.27)
Japanese vs. NHPI/Other	0.31 (0.08, 0.54)	0.14 (-0.10, 0.37)	0.05 (-0.18, 0.28)	0.06 (-0.18, 0.30)
Complete case analysis				
Chinese vs. Filipino	0.49 (0.30, 0.69)	0.39 (0.15, 0.62)	0.05 (-0.14, 0.24)	-0.00 (-0.24, 0.23)
Chinese vs. Japanese	-0.36 (-0.54, -0.19)	-0.22 (-0.42, -0.02)	0.04 (-0.14, 0.21)	0.03 (-0.17, 0.23)
Chinese vs. NHPI/Other	-0.07 (-0.27, 0.12)	-0.21 (-0.44, 0.02)	0.08 (-0.12, 0.28)	0.01 (-0.22, 0.24)
Filipino vs. Japanese	-0.86 (-1.08, -0.64)	-0.61 (-0.88, -0.34)	-0.01 (-0.23, 0.21)	0.03 (-0.24, 0.30)
Filipino vs. NHPI/Other	-0.57 (-0.81, -0.32)	-0.60 (-0.88, -0.32)	0.03 (-0.21, 0.27)	0.01 (-0.27, 0.29)
Japanese vs. NHPI/Other	0.29 (0.06, 0.52)	0.01 (-0.25, 0.28)	0.04 (-0.19, 0.27)	-0.02 (-0.28, 0.25)

Note: Standardized mean differences (SMD) were derived from multiple imputed or complete case analysis of covariance tests. Model 1 adjusted for baseline age, gender, education, and cohort. Model 2 included Model 1 and additionally adjusted for nativity and first language.

Table S3: Longitudinal associations of Asian American subgroup with cognitive function over time

	Executive function		Verbal episodic memory	
	Model 1	Model 2	Model 1	Model 2
Subgroup	β (95% CI)	β (95% CI)	β (95% CI)	β (95% CI)
Multiple imputation analysis				
Chinese	Reference	Reference	Reference	Reference
Filipino	-0.04 (-0.07, -0.01)	-0.04 (-0.07, -0.01)	-0.02 (-0.06, 0.02)	-0.02 (-0.06, 0.02)
Japanese	-0.03 (-0.05, -0.00)	-0.03 (-0.06, -0.00)	-0.03 (-0.07, 0.01)	-0.03 (-0.07, 0.01)
NHPI/Other	-0.02 (-0.05, 0.02)	-0.01 (-0.05, 0.02)	0.00 (-0.05, 0.05)	0.00 (-0.05, 0.05)
Complete case analysis				
Chinese	Reference	Reference	Reference	Reference
Filipino	-0.03 (-0.05, -0.00)	-0.04 (-0.07, -0.01)	-0.02 (-0.06, 0.02)	-0.02 (-0.07, 0.03)
Japanese	-0.02 (-0.05, 0.02)	-0.04 (-0.07, -0.01)	-0.03 (-0.07, 0.01)	-0.04 (-0.08, 0.00)
NHPI/Other	-0.04 (-0.07, -0.01)	-0.02 (-0.06, 0.01)	0.00 (-0.05, 0.05)	-0.01 (-0.06, 0.04)

Note: Estimates are for group-by-time interactions derived from linear mixed-effect models with random intercepts using multiple imputation or complete case analysis. Model 1 adjusted for baseline age, gender, education, practice effects, interview mode, and cohort. Model 2 included Model 1 and additionally adjusted for nativity and first language.

Table S4: Cohort-stratified associations of Asian American subgroup with cognitive function over time

			KHANDLE	LifeAfter90
Outcome	Term		β (95% CI)	β (95% CI)
Executive function				
	Subgroup	Chinese	Reference	Reference
		Filipino	-0.33 (-0.51, -0.15)	-0.42 (-0.67, -0.16)
		Japanese	0.39 (0.21, 0.58)	0.19 (-0.01, 0.39)
		NHPI/Other	-0.12 (-0.32, 0.08)	0.15 (-0.09, 0.39)
	Time (years)		-0.05 (-0.07, -0.03)	-0.01 (-0.04, 0.02)
	Subgroup by time	Chinese	Reference	Reference
		Filipino	-0.02 (-0.05, 0.01)	-0.11 (-0.18, -0.05)
		Japanese	0.01 (-0.03, 0.04)	-0.07 (-0.11, -0.03)
		NHPI/Other	0.00 (-0.03, 0.04)	-0.04 (-0.10, 0.02)
Verbal episodic memory				
	Subgroup	Chinese	Reference	Reference
		Filipino	-0.03 (-0.22, 0.17)	-0.00 (-0.27, 0.26)
		Japanese	0.04 (-0.15, 0.24)	-0.03 (-0.23, 0.18)
		NHPI/Other	-0.14 (-0.35, 0.07)	-0.01 (-0.26, 0.24)
	Time (years)		-0.10 (-0.14, -0.07)	-0.05 (-0.09, -0.01)
	Subgroup by time	Chinese	Reference	Reference
		Filipino	-0.01 (-0.06, 0.04)	-0.03 (-0.12, 0.07)
		Japanese	0.01 (-0.04, 0.06)	-0.10 (-0.16, -0.04)
		NHPI/Other	-0.00 (-0.06, 0.05)	0.05 (-0.04, 0.13)

Note: Estimates are derived from linear mixed-effect models with random intercepts adjusted for baseline age, gender, education, practice effects, and interview mode.
